# Single-cell and transcriptomic analyses reveal the influence of diabetes on ovarian cancer

**DOI:** 10.1186/s12864-023-09893-2

**Published:** 2024-01-02

**Authors:** Zhihao Zhao, Qilin Wang, Fang Zhao, Junnan Ma, Xue Sui, Hyok Chol Choe, Peng Chen, Xue Gao, Lin Zhang

**Affiliations:** 1 Institute (College) of Integrative Medicine, Dalian Medical University, Dalian, China; 2 Department of Pathology, the First Hospital of Dalian Medical University, Dalian, Liaoning Province 116027 China; 3 Institute of Innovation and Applied Research in Chinese Medicine, Department of Rheumatology of The First Hospital, Hunan University of Chinese Medicine, Changsha, Hunan China; 4 Department of Clinical Medicine, Sinuiju Medical University, Sinuiju, Democratic People’s Republic of Korea

**Keywords:** Ovarian cancer, Diabetes Mellitus, Single-cell RNA sequencing, Monocyte marker genes, Immunotherapy

## Abstract

**Background:**

There has been a significant surge in the global prevalence of diabetes mellitus (DM), which increases the susceptibility of individuals to ovarian cancer (OC). However, the relationship between DM and OC remains largely unexplored. The objective of this study is to provide preliminary insights into the shared molecular regulatory mechanisms and potential biomarkers between DM and OC.

**Methods:**

Multiple datasets from the GEO database were utilized for bioinformatics analysis. Single cell datasets from the GEO database were analysed. Subsequently, immune cell infiltration analysis was performed on mRNA expression data. The intersection of these datasets yielded a set of common genes associated with both OC and DM. Using these overlapping genes and Cytoscape, a protein‒protein interaction (PPI) network was constructed, and 10 core targets were selected. Gene Ontology (GO) and Kyoto Encyclopedia of Genes and Genomes (KEGG) enrichment analyses were then conducted on these core targets. Additionally, advanced bioinformatics analyses were conducted to construct a TF-mRNA-miRNA coregulatory network based on identified core targets. Furthermore, immunohistochemistry staining (IHC) and real-time quantitative PCR (RT-qPCR) were employed for the validation of the expression and biological functions of core proteins, including HSPAA1, HSPA8, SOD1, and transcription factors SREBF2 and GTAT2, in ovarian tumors.

**Results:**

The immune cell infiltration analysis based on mRNA expression data for both DM and OC, as well as analysis using single-cell datasets, reveals significant differences in mononuclear cell levels. By intersecting the single-cell datasets, a total of 119 targets related to mononuclear cells in both OC and DM were identified. PPI network analysis further identified 10 hub genesincludingHSP90AA1, HSPA8, SNRPD2, UBA52, SOD1, RPL13A, RPSA, ITGAM, PPP1CC, and PSMA5, as potential targets of OC and DM. Enrichment analysis indicated that these genes are primarily associated with neutrophil degranulation, GDP-dissociation inhibitor activity, and the IL-17 signaling pathway, suggesting their involvement in the regulation of the tumor microenvironment. Furthermore, the TF-gene and miRNA-gene regulatory networks were validated using NetworkAnalyst. The identified TFs included SREBF2, GATA2, and SRF, while the miRNAs included miR-320a, miR-378a-3p, and miR-26a-5p. Simultaneously, IHC and RT-qPCR reveal differential expression of core targets in ovarian tumors after the onset of diabetes. RT-qPCR further revealed that SREBF2 and GATA2 may influence the expression of core proteins, including HSP90AA1, HSPA8, and SOD1.

**Conclusion:**

This study revealed the shared gene interaction network between OC and DM and predicted the TFs and miRNAs associated with core genes in monocytes. Our research findings contribute to identifying potential biological mechanisms underlying the relationship between OC and DM.

**Supplementary Information:**

The online version contains supplementary material available at 10.1186/s12864-023-09893-2.

## Introduction

OC is as the primary contributor to mortality among malignant tumors affecting the female reproductive system, leading to a global toll of 207,252 deaths [1]. Conventional therapy, including cytoreductive surgery and chemotherapy, has a 90% effectiveness rate when the cancer is diagnosed at an early stageand confined to one or both ovaries. However, the majority of ovarian cancer cases are diagnosed at stage III or IV, when the cancer has metastasized, and the 5-year survival rate for these patients is 30% [2]. Recently, the tumor microenvironment and tumor immunology of OC have become research hotspots [3]. Factors such as tumor-related inflammation [4], angiogenesis [5], and immune evasion [6] play critical roles in the development and progression of OC. Peripheral blood mononuclear cells (PBMCs) have been widely utilized in DM research [7]. As a population of immune cells in peripheral blood, PBMCs consist of various cell types, including lymphocytes, monocytes, and natural killer cells [8]. Comprehensive analysis of PBMCs from diabetic patients enables exploration of key features such as immune-metabolic dysregulation, inflammatory responses [9], and cellular functional changes associated with diabetes [10]. Given the current fatality rate of OC and the prevalence of diabetes, researchers and clinicians are increasingly interested in investigating whether the presence of DM in OC patients contributes to further disease exacerbation and poorer outcomes for both OC and DM.

Among all cancer patients, 8–18% have diabetes [11]. Particularly, the risk of ovarian cancer is significantly increased in women with diabetes [12], and patients with epithelial ovarian cancer and concomitant diabetes exhibit a much lower overall survival rate compared to non-diabetic patients [13]. Insulin can induce apoptosis in ovarian cancer cells through cell cycle regulation and influence inflammation and immune response [14]. Moreover, extensive research suggests that anti-diabetic medications like metformin can significantly inhibit the occurrence and development of ovarian cancer [15]. Simultaneously, potential hyperglycemia has the potency to promote ovarian cancer formation. Elevated glucose levels accelerate the growth of ovarian tumors in a glucose concentration-dependent manner and significantly shorten overall survival [16]. Ovarian cancer cells also exhibit high glycolytic activity, and normal circulating glucose concentrations may not meet the energy demands of the tumor, potentially acting as a limiting factor in cancer cell metabolism. Elevated blood glucose in diabetic patients may fulfill these energy demands, thus promoting cancer progression. There is evidence to suggest that elevated insulin-like growth factor-1 (IGF-1) levels in diabetic patients lead to an increase in cytokine and estrogen levels, an imbalance in adipokines, and hyperinsulinemia, thereby increasing the risk of ovarian cancer and impacting patients’ survival [17, 18]. Furthermore, various proteins involved in glucose metabolism, participating in glucose metabolism in diabetic patients, have been identified as potential therapeutic targets for OC treatment. Chronic hyperglycemia may lead to alterations in the ovarian cancer microenvironment, including increased angiogenesis and tumor immune evasion, promoting the progression of ovarian cancer. Therefore, it is essential to further investigate potential biomarkers associated with OC and reveal the possible mechanisms and common therapeutic targets in monocyte cells between OC and DM.

**Fig. 1:**
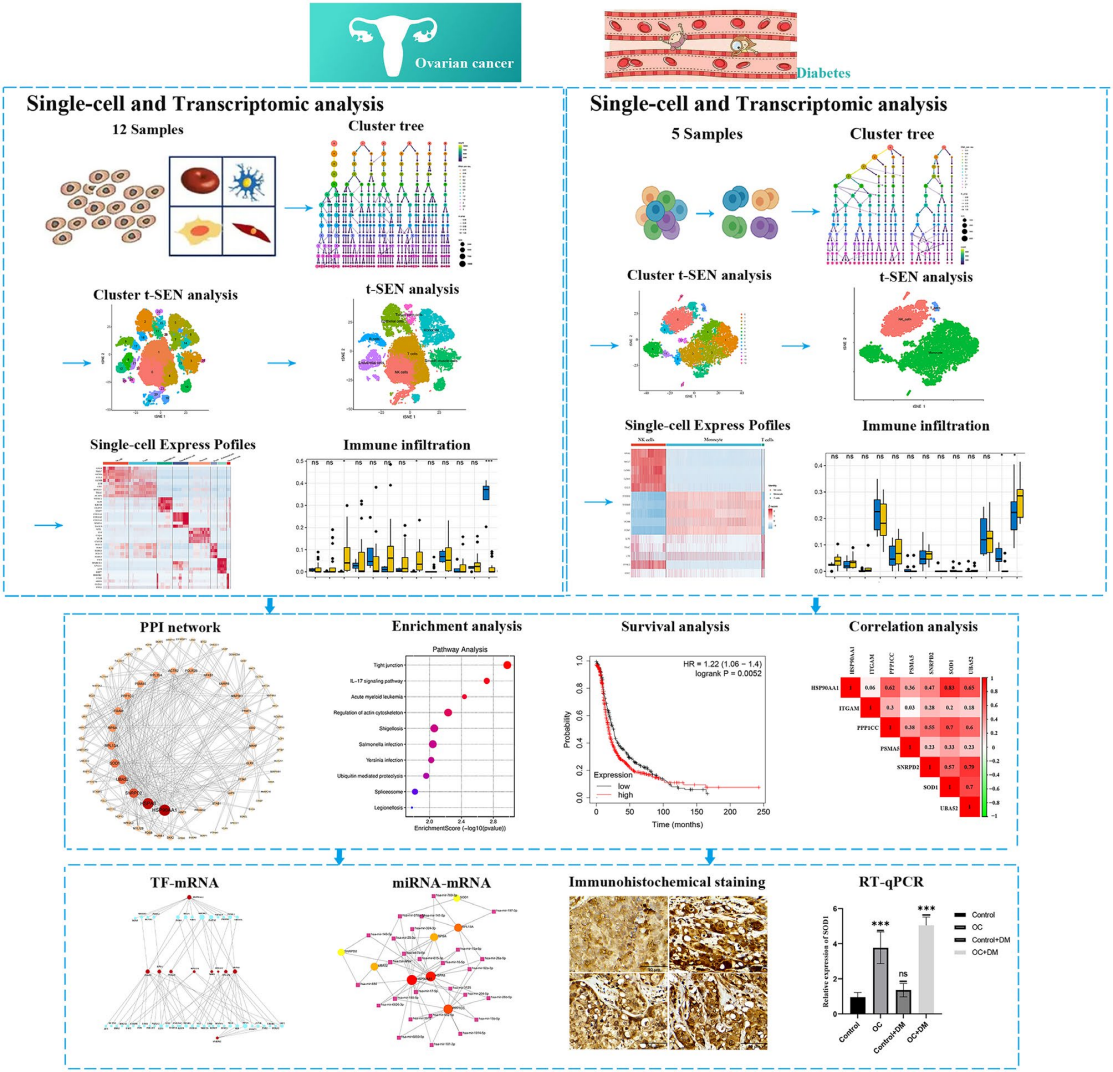
Flow chart of study design. PPI, protein‒protein interaction; TF, transcription factor; miRNA, microRNA; RT-qPCR, real time quantitative polymerase chains reaction

The advent of single-cell RNA sequencing (scRNA-seq) technology and its associated data analysis methods has presented an unprecedented opportunity to unravel the molecular characteristics of diverse immune cell populations within the tumor microenvironment (TME) [19]. Previous studies have revealed that exploring gene expression signatures based on molecular characteristics of immune cells derived from scRNA-seq data can be a powerful approach to predicting the prognosis and response to immunotherapy in cancer patients [20, 21]. In this study, we analyzed scRNA-seq data (GSE184880 and GSE165816) from OC and DM patients, revealing significant differential expression in monocytes between the two disease models, with a larger proportion of monocytes in PBMCs from diabetic patients. Additionally, we downloaded mRNA data for OC and DM from the GEO database (GSE40595 and GSE29142) and performed immune infiltration analysis, showing shared immune cell infiltrations between OC and diabetic samples, including plasma cells, follicular helper T cells, monocytes, resting mast cells, and neutrophils. Further investigation involved screening 119 common differentially expressed genes related to monocytes from OC and DM scRNA-seq data, visualizing their expression levels in a heatmap. By constructing a PPI network and analyzing the connections between these targets, we identified 10 hub genes based on their topological importance. Subsequently, enrichment analysis using KEGG and GO was conducted to elucidate the biological functions associated with these central genes. After analyzing the 10 core genes, we compared their differential expression in 426 ovarian cancer tissues and 88 normal tissues from the TCGA database. Additionally, survival analysis was performed on 146 ovarian cancer patients from the TCGA database for these 10 core genes. Pearson correlation analysis was conducted on several hub genes from GSE40595 and GSE29142. Finally, gene regulatory network analysis was performed to identify key TFs and miRNAs enriched in the hub genes. Validation of core genes and TFs was conducted through immunohistochemical staining and RT-qPCR. In summary, our study provides new insights that can help understand the potential mechanisms and common therapeutic targets between OC and DM (Fig. 1).

## Materials and methods

### Data capture from single-cell data

Data for OC and DM were obtained from the Gene Expression Omnibus (GEO) database, a repository of scRNA-seq data maintained by the National Center for Biotechnology Information (NCBI) (https://www.ncbi.nlm.nih.gov/geo/). The scRNA-seq dataset GSE184880 [22], which consisted of 7 untreated ovarian cancer patients with early or advanced tumor stages and 5 age-matched nonmalignant ovarian samples, was selected based on the GPL24676 Illumina NovaSeq 6000 human genome microarray study. For diabetic scRNA-seq, the dataset GSE165816 [23] was downloaded from the GEO platform. The GPL24676 protocol was employed for single-cell RNA-seq analysis of foot and forearm skin samples, as well as PBMC samples, from a cohort of 10 nondiabetic individuals and 17 individuals with diabetes.

### Dimensionality reduction analysis and cell subpopulation identification of single-cell data

To ensure the accuracy of cell subpopulations, we utilized the Seurat R tool for data analysis. Normal and diseased tissues were merged into a single Seurat object. We applied the “LogNormalize” method to normalize the Seurat object, with the scale factor set to 10,000. Single-cell data were filtered based on the following criteria: Cells expressing fewer than 200 genes and fewer than three genes were excluded. Cells expressing 200 to 8000 genes and less than 20% mitochondrial genes were retained. *P*-values were calculated using the default Wilcoxon Rank Sum test through the FindMarkers function. Differentially expressed genes were determined by Bonferroni and adjusted p-values were computed using the Benjamini-Hochberg (BH) method, with a threshold set at or below 0.05. Subsequently, highly variable genes were identified through data normalization using log normalization and the FindVariableFeatures tool. Principal component analysis (PCA) was applied to reduce dimensionality after scaling the data using the ScaleData tool. The cells were clustered by employing the FindNeighbors and FindClusters functions (with a resolution set to 1.2) to identify cellular subgroups. Subsequently, different cell clusters were determined and annotated based on the composition pattern of marker genes using the single R package. Finally, manual validation and correction were performed using the CellMarker database.

### Integration of microarray data

Human mRNA expression data from ovarian cancer (GSE40595) and diabetes (GSE29142) studies were obtained from the GEO database. GSE40595 [24] utilized the Affymetrix Human Genome U133 Plus 2.0 Array platform to analyze gene expression profiles of 77 samples, including stromal and epithelial components from 63 ovarian cancer patients, as well as gene expression profiles of 14 normal ovarian stromal and epithelial samples. Additionally, GSE29142 [25] employed the Phalanx Human OneArray platform to examine 19 samples, comprising 10 healthy control samples without diabetes and 9 PBMC samples from individuals with diabetes. All data analyses were conducted in R 4.2.3, and boxplots were generated using ggplot2.

### Screening for shared genes in mononuclear cells and immune cell infiltration

Differential gene expression analysis was performed on two datasets by comparing expression values across different groups using the linear modeling module of Bioconductor for microarray data [26]. Normalization and log_2_ transformation were applied to each dataset, and differentially expressed genes (DEGs) were identified. The most significant genes were visualized using heatmaps. CIBERSORT, a deconvolution algorithm [18], was utilized to describe the cellular composition of tissues based on the gene expression patterns. To determine the abundance of immune cells in PBMCs of OC patients and diabetic patients, the LM22 matrix was used as a reference [27]. The output of CIBERSORT, which assesses the reliability of results for all samples, was generated using Monte Carlo sampling [28]to obtain deconvoluted P values. Furthermore, to elucidate the interplay between DM and OC, we screened for common genes in monocytes between the OC-associated targets and DM-associated targets.

### Construction of protein‒protein interaction networks and identification of hub genes

PPIs represent a major component of cellular biochemical reaction networks. Evaluating PPI networks and understanding their functions is a critical objective for gaining insights into cellular machinery processes in both cellular and molecular systems biology [29].To construct the Protein‒Protein interaction network, the STRING database (https://cn.string-db.org/) was utilized. The following parameters were set: in the network display options, disconnected nodes were hidden. To obtain more comprehensive protein interaction information and generate a more complex network, the minimum required interaction score was set to a moderate confidence level of 0.4. The data were imported into Cytoscape 3.8.2 software to generate the gene network diagram. Subsequently, network topological analysis was performed using the Cytoscape plugin “cytoHubba”. The top ten nodes were selected based on their degree ranking for further analysis.

### Functional enrichment analysis

GO is a prominent bioinformatics tool used for annotating genes and analyzing the biological processes associated with these genes. GO enrichment analysis of molecular function (MF), cellular components (CC), and biological process (BP) categories reveals overrepresented or underrepresented GO terms within a given set of genes [30]. KEGG is a major database resource that allows the understanding of high-level functions and biological systems based on large-scale molecular datasets generated from high-throughput experimental techniques [31]. Therefore, the R programming language packages ‘clusterProfiler’, ‘org.hs.eg.db’, ‘EnrichPlot’, and ‘ggplot2’ were employed to conduct GO and KEGG enrichment analysis on the shared targets of the aforementioned mononuclear cells, facilitating further analysis of the common genes [32, 33].

### Evaluation of crucial genes correlations and survival analyses

Survival analysis entails the methodological approach of analyzing and inferring the survival time of organisms or individuals based on data acquired from experiments or surveys. It involves studying the relationship between survival time, outcomes, various influencing factors, and their magnitudes [34]. The GEPIA database is an online platform for interactive analysis of gene expression profiles, which is an RNA sequencing data platform (http://gepia.cancer-pku.cn/). It incorporates data from 9,736 tumor tissues and 8,587 normal tissues from both TCGA and GTEx databases [35]. By navigating to the “Box Plots” module in the GEPIA database, one can retrieve the differential expression of the top ten core genes in ovarian cancer compared to normal tissues.Utilizing the TCGA database (https://kmplot.com/analysis/), Kaplan-Meier analysis was conducted to evaluate the prognostic value of the top ten core genes between high and low expression groups. Univariate Cox regression analysis was performed to examine the relationship between the expression of the top ten core genes and overall survival (OS), adjusting for age and tumor stage.Subsequently, Pearson correlation coefficient analysis was employed to investigate the interrelations among the core genes in different sample types. The Pearson correlation coefficient (r) values were calculated using the “corrplot” package in the R programming language, and a heatmap was generated for visualization.

### Identification of pivotal gene-associated TFs and miRNAs

NetworkAnalyst is an online analysis platform for gene expression profiling and curation analysis that integrates advanced statistical methods and innovative data visualization systems. It enables differential analysis, functional analysis, and network analysis of differential analysis results [36, 37]. TFs are proteins that bind to specific DNA sequences and control gene expression, making them crucial for molecular understanding [38]. We obtained the core gene-TF network topology graph through ENCODE. Additionally, miRNAs negatively affect protein expression by destabilizing the stability and translation efficiency of target mature messenger RNAs. Therefore, we analyzed the core gene-miRNA network topology graph using MiRTarBase [39]. Ultimately, Cytoscape was utilized to visualize the networks depicting the interactions between TF and genes and miRNA and genes.

### Immunohistochemical staining

To validate the protein levels of key genes in ovarian tumor samples, we selected 4 cases of non-malignant ovarian samples and 4 cases of untreated high-grade serous ovarian cancer samples. Additionally, there were 4 cases of non-malignant ovarian samples combined with diabetes and 4 cases of high-grade serous ovarian cancer samples combined with diabetes. The study has been approved by the Ethics Committee of the First Affiliated Hospital of Dalian Medical University. Ovarian tumor samples, after fixation in formalin and paraffin embedding, were sectioned, deparaffinized in xylene, and hydrated in graded ethanol. Subsequently, antigen retrieval was performed in 100 °C citrate buffer, followed by peroxidase blocking of the sections. The sections were then washed with PBS. The slices were incubated with primary antibodies against HSP90AA1 (1:100, rabbit, proteintech, 10654-1-AP) and HAPA8 (1:100, rabbit, proteintech, 13171-1-AP) overnight at 4 °C. After washing with PBS, the sections were incubated with enzyme-labeled goat anti-mouse/rabbit IgG polymer, stained with 3,3’-diaminobenzidine (DAB), and counterstained with hematoxylin. The samples were dehydrated, covered with cover slips, and images were captured using a Leica Microscope Imaging System (Leica, DE).

### RNA isolation and reverse transcription-quantitative polymerase chain reaction

Total RNA was isolated from ovarian tumor tissues using RNA extraction reagent (Amoy Diagnostics). cDNA was reverse transcribed using a reverse transcriptase kit (Promega). Subsequently, amplification reactions were performed using a reaction system composed of 10 µl SYBR-Green qPCR Master Mix, 1 µl cDNA template, 7.8 µl DEPC water, and 0.6 µl each of forward and reverse primers. The thermal cycling conditions for the PCR reaction were as follows: an initial denaturation at 95 °C for 2 min, followed by denaturation at 95 °C for 15 s, annealing at 59 °C for 20 s, and extension at 60 °C for 40 s. GAPDH was used as the reference gene, and the relative expression level was calculated using the 2-ΔΔCt method. The primer sequences for the target genes and the reference gene were as follows: HSP90AA1, forward, 5ʹ-TAT AAG GCA GGC GCG GGG GT-3ʹ, reverse, 5ʹ-TGC ACC AGC CTG CAA AGC TTC C-3ʹ; HSPA8, forward, 5ʹ-TTG CTG CTC TTG GAT GTC-3ʹ, reverse, 5ʹ-TGT GTC TGC TTG GTA GGA-3ʹ; SOD1, forward, 5ʹ-GCC GAT GTG TCT ATT GAA G-3ʹ, reverse, 5ʹ-AGC GTT TCC TGT CTT TGT-3ʹ; SREBF2, forward, 5ʹ-GGA GAC CAG GAA GAA GAG A-3ʹ, reverse, 5ʹ-CAC CAC CGA CAG ATG ATG-3ʹ; GATA2, forward, 5ʹ-ACG ACA ACC ACC ACC TTA-3ʹ, reverse, 5ʹ-TTC TTG CTC TTC TTG GAC TT-3ʹ; GAPDH, forward, 5′- TAT GAC AAC AGC CTC AAG AT-3′, reverse, 5′- AGT CCT TCC ACG ATA CCA-3′.

### Statistical analysis

The calculations and statistical analyses were performed using R 4.2.2 software and GraphPad Prism 9. The comparison between two groups was conducted using the Wilcoxon rank-sum test, while the Kruskal‒Wallis test was employed for comparisons involving more than two groups. Survival analysis was carried out using the Kaplan‒Meier method with log-rank test. The correlation analysis of key genes was performed using the Pearson method. *p* < 0.05 was considered to indicate statistical significance.

## Results

### Identification of the gene expression profile of monocytes in ovarian cancer

**Fig. 2:**
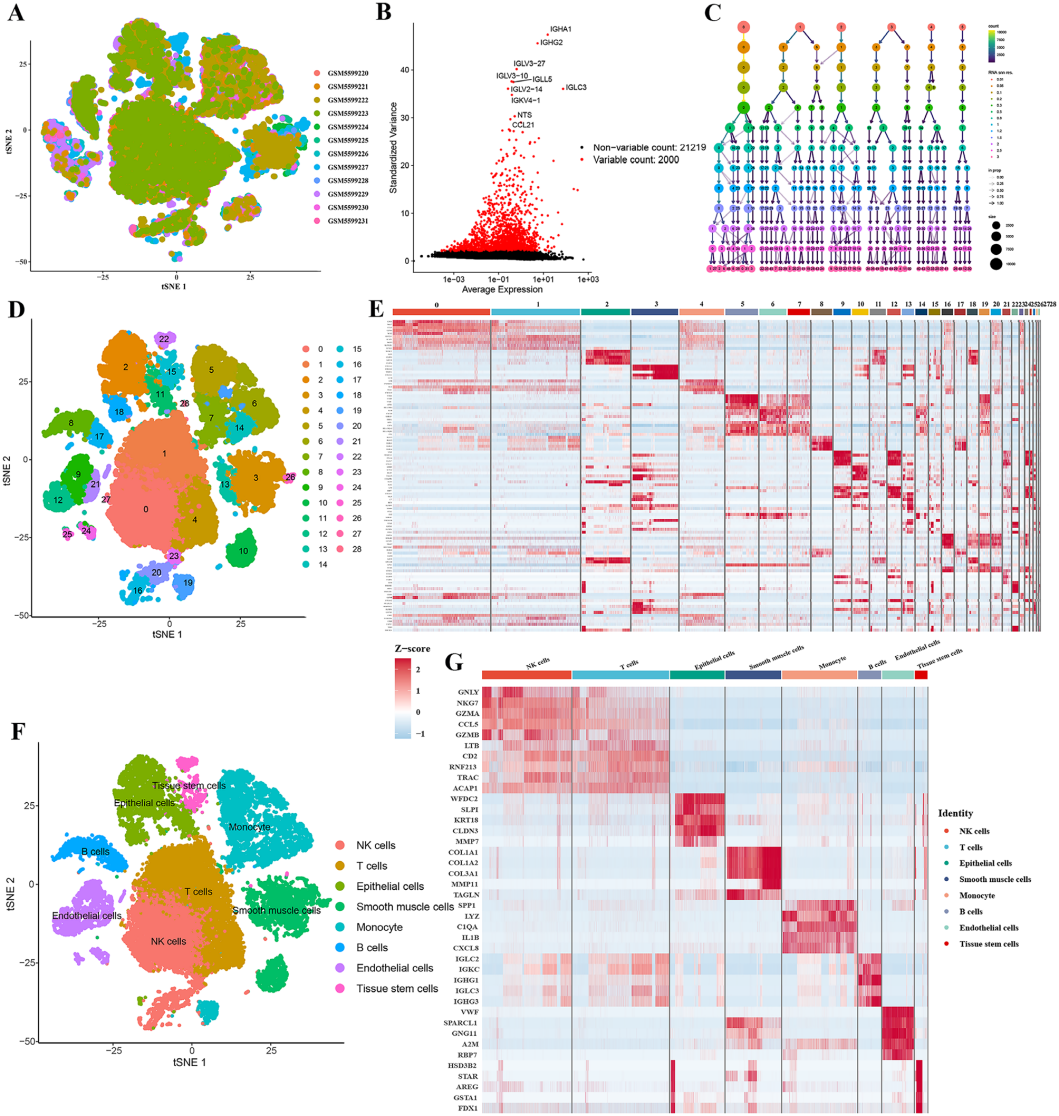
Identification of OC Subtypes. **(A)** t-SNE plot of 12 samples from the GSE184880 dataset after dimensionality reduction and batch correction; **(B)** top 10 differentially expressed genes in ovarian cancer; **(C)** Seurat clustering results with resolutions ranging from 0 to 3, where different colors represent different resolutions and larger dots indicate a higher number of cells in the subgroups; **(D)** t-SNE plot of the 29 cell clusters classified based on scRNA-seq data; **(E)** heatmap showing the relative expression levels of marker genes in the 29 cell clusters; **(F)** t-SNE plot indicating the identification of various cell subtypes; **(G)** heatmap displaying the relative expression levels of marker genes in different cell subtypes

The scRNA-seq data of ovarian cancer used in this study were obtained from 59,324 cells from tumor samples in the GSE184880 dataset. After logarithmic normalization, the single-cell sequencing dataset of OC samples was analyzed, revealing good integration across 12 samples with no apparent batch effects (Fig. 2A), making it suitable for subsequent analysis. We selected the top 2,000 highly variable genes and annotated the top 10 highly variable genes simultaneously (Fig. 2B). The “FindNeighbors” and “FindClusters” functions from the “Seurat” package were used to perform unsupervised clustering analysis on the filtered cells, with a resolution ranging from 0.01 to 3. PCA was used for dimensionality reduction, and 16 PCs with a p value < 0.05 were selected for further analysis (Fig. 2C). Ultimately, we obtained 29 clusters, which were visualized using t-SNE plots (Fig. 2D). From these 29 clusters, a total of 28,466 differentially expressed marker genes were identified (Table S1), and the relative expression levels of these marker genes in each cluster are displayed in a heatmap (Fig. 2E). Subsequently, with reference to known cell type marker genes in the CellMarker database, we annotated these cell clusters using the Single R algorithm, ultimately identifying eight cell types: NK cells, T cells, epithelial cells, smooth muscle cells, monocytes, B cells, endothelial cells, and tissue stem cells (Fig. 2F). Clusters 5, 6, 7, 14, and 19 were annotated as monocytes. Among the annotated cell clusters, a total of 16,617 differentially expressed genes were identified (Table S2), and the relative gene expression levels in each cell cluster were visualized in a heatmap (Fig. 2G).

### Identification of the monocyte cell population in the gene expression atlas of Diabetes using scRNA-seq

**Fig. 3:**
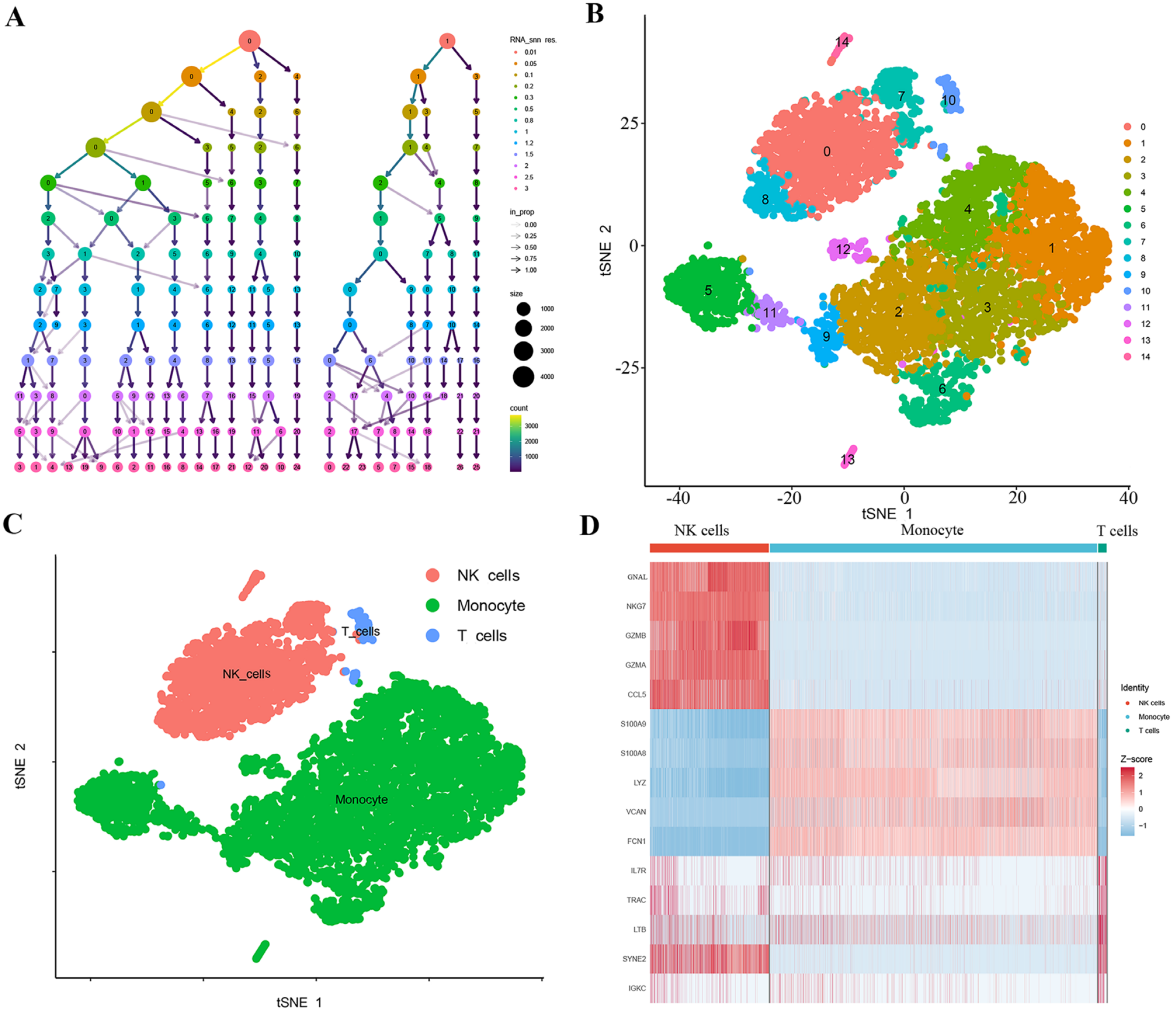
PBMC Gene Expression Profiles of Monocytes in Diabetic Peripheral Blood. **(A)** Seurat clustering results with a resolution ranging from 0 to 3. Different colors represent different resolutions, and larger dots indicate subpopulations with a higher number of cells. **(B)** t-SNE plots illustrating the classification of 15 cell clusters based on scRNA-seq data. **(C)** t-SNE plots used for identifying distinct cell subtypes. **(D)** Heatmap displaying the relative expression of marker genes in various cell subtypes

The PBMC scRNA-seq data GSE165816 for diabetes were downloaded from the official GEO website, and the selected samples are shown in Table 1. The data preprocessing steps: including cell filtering using the “Seurat” package to remove low-quality cells, logarithmic normalization, and selection of the top 2,000 highly variable genes with their top ten labeled as highly variable genes. Unsupervised clustering analysis was performed on the filtered cells, and an appropriate resolution was chosen. PCA was employed for dimensionality reduction, and 14 PCs with a p value < 0.05 were selected for further analysis (Fig. 3A). Ultimately, 15 clusters were obtained and visualized using t-SNE (Fig. 3B). From these clusters, a total of 5,875 differentially expressed marker genes were identified (Table S3). Subsequently, using reference marker genes available in the CellMarker database, the cell clusters were annotated using the Single R algorithm, resulting in the identification of three cell types: NK cells, monocytes, and T cells (Fig. 3C). Among these, Clusters 1, 2, 3, 4, 5, 6, 9, 11, 12, and 13 were annotated as monocytes. A total of 4,161 differentially expressed genes were identified within the annotated cell clusters (Table S4), and the relative gene expression of each cell cluster was visualized in the form of a heatmap (Fig. 3D).

**Table 1:** Clinical data for single cell analysis of PBMC from diabetic and nondiabetic patients

Sample	DM 1	DM 2	Normal 1	Normal 2	Normal 3
Age (years)	58	68	56	62	74
BMI (kg/m²)	25.98	34.40	25.69	31.62	25.99
Blood Urea Nitrogen (mg/dL)	-	18	11	18	16
Creatinine (mg/dL)	0.9	1.3	0.7	0.7	0.8
Cholesterol, Total (mg/dL)	180	142	179	144	153
Triglycerides (mg/dL)	124	241	76	108	169
LDL Cholesterol (mg/dL)	77	78	97	78	35

### Immune cells infiltrating both OC and DM samples

**Fig. 4:**
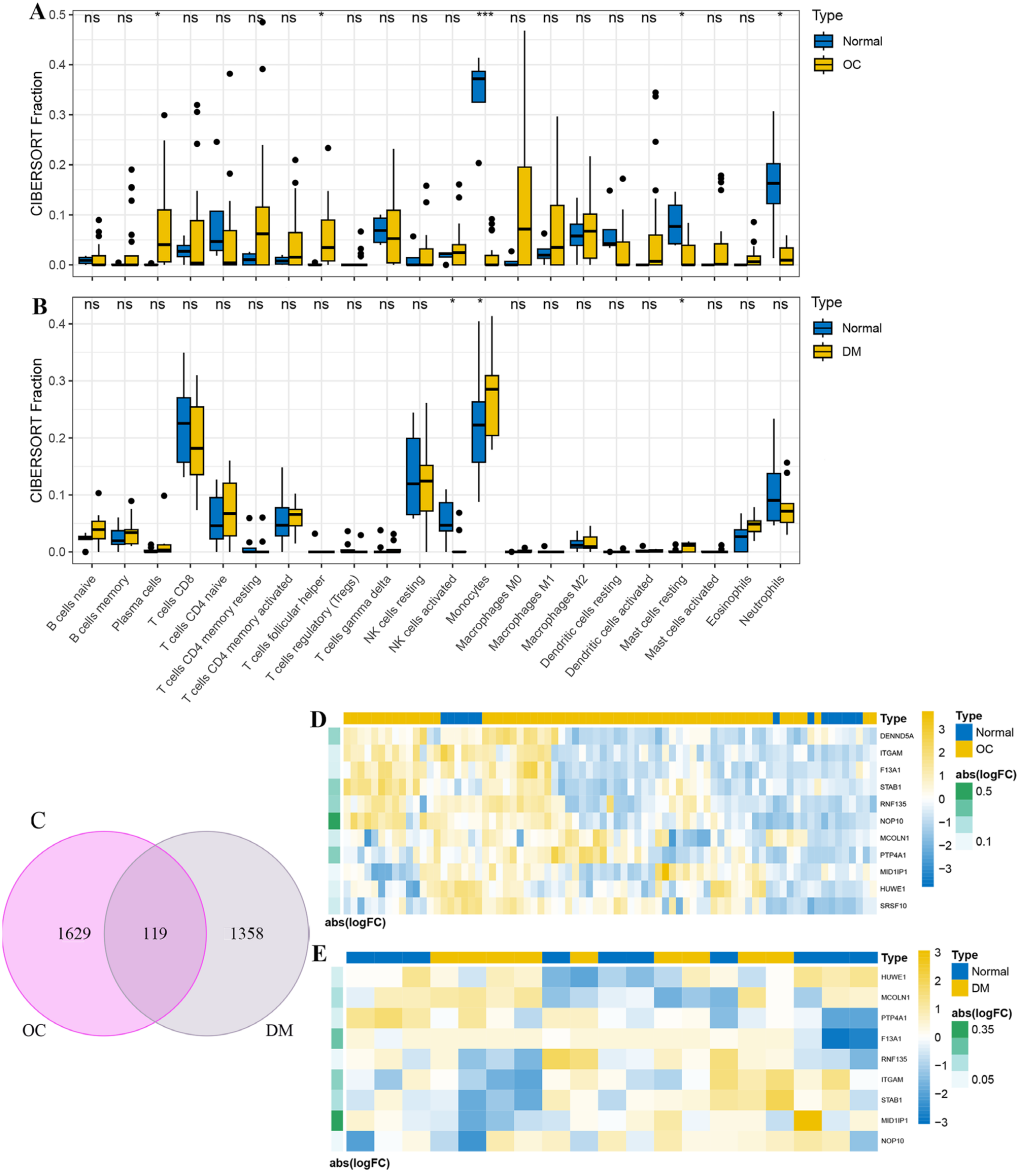
Simultaneous involvement of mononuclear cells in both DM and OC pathogenesis. **(A)** Immune cell composition analysis using CIBERSORT in GSE40595; **(B)** immune cell composition analysis using CIBERSORT in GSE29142. The x-axis represents immune cell types, while the y-axis represents the relative abundance of different samples; **(C)** Venn diagram demonstrating the overlapping genes among differentially expressed genes in monocytes between GSE184880 and GSE165816. A total of 119 common genes were identified. **(D)** Heatmap depicting the intersection of differentially expressed genes in GSE40595 with the 119 common genes; **(E)** heatmap illustrating the intersection of differentially expressed genes in GSE29142 with the 119 common genes. Each column represents a specific gene, while each row corresponds to a sample or condition. Data were presented as the mean ± SD. *p < 0.05, **p < 0.01, *** *p* < 0.001

CIBERSORT analysis was conducted on human mRNA expression data from the GSE40595 and GSE29142 studies to investigate immune cell infiltration and establish the correlation between OC samples and diabetic samples, involving 22 immune cell types. The analysis revealed that OC samples were similar to normal samples in terms of infiltrating immune cell types, which included plasma cells, T cells follicular helper, monocytes, mast cells resting, and neutrophils (Fig. 4A; *p* < 0.05, *p* < 0.01, *p* < 0.001). Moreover, diabetic peripheral blood PBMC samples showed infiltration patterns similar to those of normal samples, particularly in terms of activated NK cells, monocytes, and resting mast cells (Fig. 4B; *p* < 0.05). Monocytes and resting mast cells were identified as immune cell infiltrates common to both OC and DM.

Monocytes originate from bone marrow cells and represent a subset of cells present in peripheral blood [40]. Both PBMC collected from blood and those existing within the tumor environment are circulating immune cells in the periphery. Monocytes predominantly circulate in the bloodstream, and there’s a remarkable similarity between the monocytes identified within the cancer stroma and those present in the bloodstream [40].To further investigate this, we compared the putative 1748 putative differentially expressed monocyte genes in OC samples with the probable 1477 monocyte genes in diabetic PBMC samples based on the findings from scRNA-seq analysis. By intersecting the differentially expressed genes, we identified a total of 119 common differentially expressed genes (Fig. 4C). Subsequently, we intersected the 119 common differentially expressed genes with the differentially expressed genes specific to OC (Fig. 4D) and DM (Fig. 4E) in the mRNA expression data. The resulting genes from this intersection were visualized in a heatmap based on their expression levels.

### Functional analysis of critical module genes

**Fig. 5:**
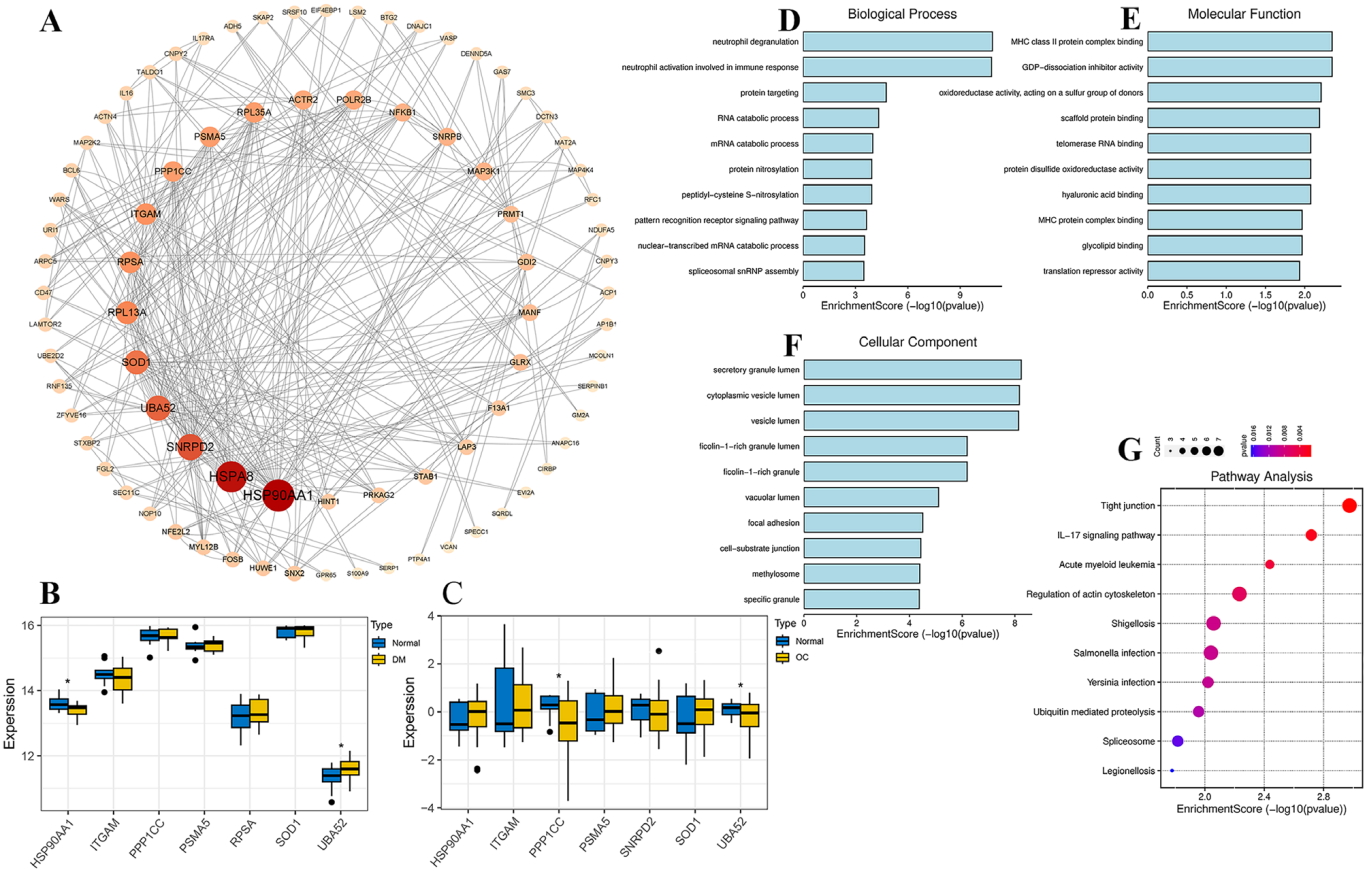
Identification and functional enrichment analysis of hub genes. **(A)** Visualization of the PPI network using Cytoscape 3.8.2 software. Each node represents a protein, and each edge represents the relationship between two proteins. The size and color intensity of a node indicate its importance in the network. **(B)** Bar plot showing the intersection of differentially expressed genes in GSE29142 with the 119 shared genes in monocytes. **(C)** Bar plot depicting the intersection of differentially expressed genes in GSE40595 with the 119 shared genes in monocytes. **(D)** GO enrichment analysis of the 119 shared genes, including biological processes. **(E)** GO enrichment analysis of the 119 shared genes, focusing on molecular functions. **(F)** GO enrichment analysis of the 119 shared genes, highlighting cellular components. **(G)** KEGG enrichment analysis of the 119 common genes involved. BP, biological process; MF, molecular function; CC, cellular component. * *p* < 0.05

The 119 duplicated genes in monocytes were uploaded to the STRING database, and the resulting free nodes were eliminated to construct the PPI network, which was visualized using Cytoscape. The PPI network consisted of 87 nodes and 328 edges (Fig. 5A). Using the cytoHubba plug-in in Cytoscape with a target connectivity threshold of 30 (triple the median value), the top 10 hub genes were identified: HSP90AA1, HSPA8, SNRPD2, UBA52, SOD1, RPL13A, RPSA, ITGAM, PPP1CC, and PSMA5. To examine the expression levels of these hub genes, a heatmap was generated using mRNA expression data. Among these genes, HSP90AA1 and UBA52 exhibited significant differences between DM samples and normal samples (Fig. 5B; *p* < 0.05), while PPP1CC and UBA52 showed significant differences between OC samples and normal samples (Fig. 5C; *p* < 0.05). The 119 common genes were subjected to GO enrichment analysis using R language. The analysis revealed the following findings (Table S5). In the biological process (BP) category, the common genes were primarily associated with neutrophil degranulation, neutrophil activation involved in immune response, protein targeting, RNA catabolic process, mRNA catabolic process, protein nitrosylation, peptidyl-cysteine S-nitrosylation, pattern recognition receptor signaling pathway, nuclear-transcribed mRNA catabolic process, and spliceosomal snRNP assembly (Fig. 5D). Under high glucose conditions, ovarian cancer cells generate an inflammatory response, leading to an elevation in neutrophil levels, while the levels of functional lymphocytes often decrease. Neutrophils induce various cytokines and contribute to angiogenesis and the growth of ovarian cancer [41]. In the molecular function (MF) category, the common genes were mainly enriched in GDP-dissociation inhibitor activity, MHC class II protein complex binding, oxidoreductase activity (acting on a sulfur group of donors), scaffold protein binding, hyaluronic acid binding, protein disulfide oxidoreductase activity, telomerase RNA binding, MHC protein complex binding, glycolipid binding, and translation repressor activity (Fig. 5E) MHC class II proteins induce apoptosis in ovarian cancer cells by presenting insulin or insulin-related antigens [42]. In the cell component (CC) category, the common genes were predominantly associated with secretory granule lumen, cytoplasmic vesicle lumen, vesicle lumen, ficolin-1-rich granule, ficolin-1-rich granule lumen, vacuolar lumen, focal adhesion, cell-substrate junction, methylosome, and specific granule (Fig. 5F). The secretory granule lumen regulates inflammation and immunity in mice and suppresses the occurrence and development of ovarian cancer [43]. Furthermore, these common genes were subjected to KEGG pathway enrichment analysis. The results indicated that the common genes were primarily involved in tight junction, the IL-17 signaling pathway, acute myeloid leukemia, regulation of actin cytoskeleton, shigellosis, Salmonella infection, Yersinia infection, ubiquitin-mediated proteolysis, spliceosome, and legionellosis (Fig. 5G, Table S6).

### Prognostic relevance of hub genes in OC

**Fig. 6:**
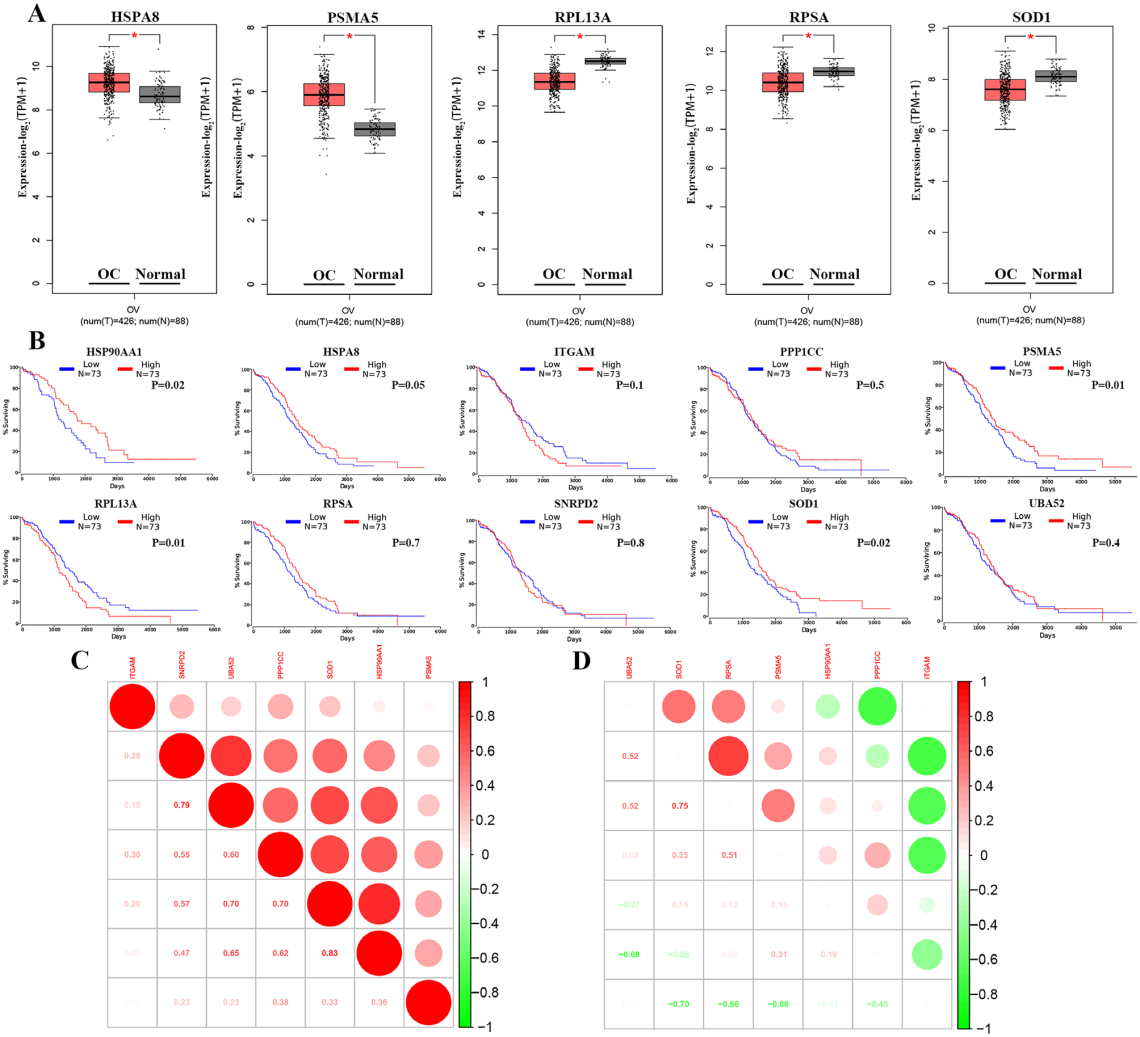
Validation of hub gene expression and survival. **(A)** mRNA expression of hub genes in ovarian cancer tissues compared to normal tissues, indicated by asterisks. **(B)** Kaplan‒Meier survival analysis of 10 hub genes in mononuclear cells of OC patients, showing high and low expression groups. **(C)** Pearson correlation analysis of hub genes in the GSE40595 dataset; **(D)** pearson correlation analysis of HUB genes in the GSE29142 dataset. The color indicates the strength of the correlation. Correlation coefficients between 0 and 1 represent positive correlation, between − 1 and 0 represent negative correlation. The larger the absolute value of the coefficient, the stronger the correlation. * *p* < 0.05

We performed an analysis of gene expression profiles in ovarian cancer samples and normal tissues using GEPIA. This analysis, based on TCGA and GTEx datasets, revealed significant differences (*p* < 0.05) in five out of the ten core genes, including HSPA8, PSMA5, RPL13A, RPSA, and SOD1. Additionally, the expression levels of HSPA8 and SOD1 were higher in high-grade serous ovarian cancer samples compared to non-malignant ovarian tumor tissues, while RPL13A, PSMA5, and RPSA showed the opposite trend (Fig. 6A). To assess the prognostic value of the selected core genes in ovarian cancer, we plotted specific survival curves using the TCGA database (Fig. 6B). Kaplan–Meier curve analysis revealed a significant correlation (*p* < 0.05) between low expression of HSP90AA1, HSPA8, PSMA5, and SOD1 and prolonged overall survival (OS) in ovarian cancer patients. Conversely, low expression of RPL13A was significantly associated with shorter OS (*p* < 0.05). However, the expression of ITGAM, PPP1CC, RPSA, and SNRPD2 showed no significant correlation with OS in OC patients (Fig. 6B). Furthermore, we conducted Pearson correlation coefficient analysis to assess the reproducibility and correlation of these ten core genes in two GEO datasets. The analysis revealed positive correlations among HSP90AA1, ITGAM, PPP1CC, PSMA5, SNRPD2, SOD1, and UBA52 in the GSE40595 dataset (Fig. 6C). Additionally, in GSE29142, we observed a negative correlation between HSP90AA1, ITGAM, PPP1CC, and other core genes. On the other hand, positive correlations were observed among PSMA5, SNRPD2, SOD1, and UBA52 (Fig. 6D).

### Construction of regulatory networks

**Fig. 7:**
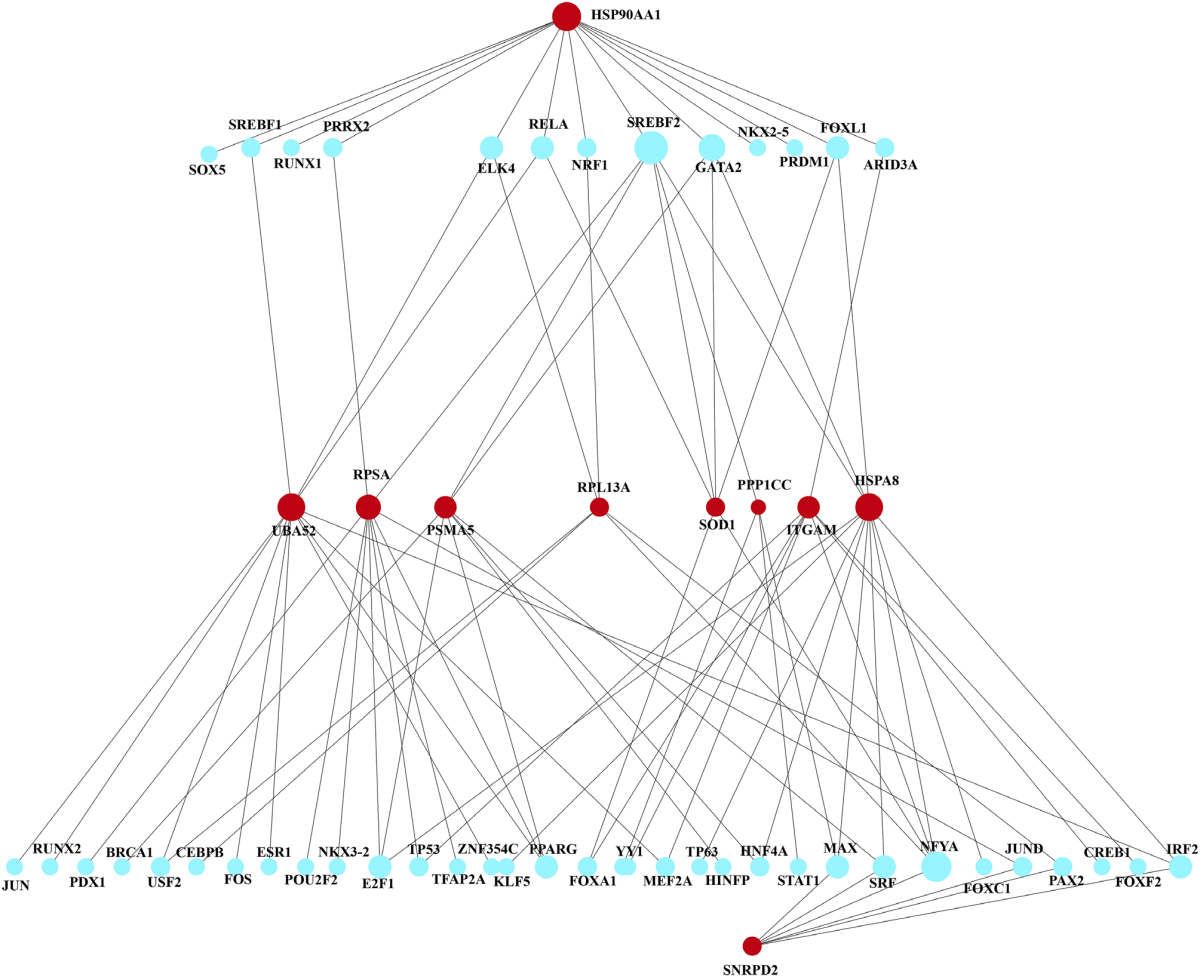
Network Analyst generated an interconnected regulatory interaction network of TF genes, in which blue circular nodes represent TFs and genes interacting with TFs are depicted as red circular nodes

**Fig. 8:**
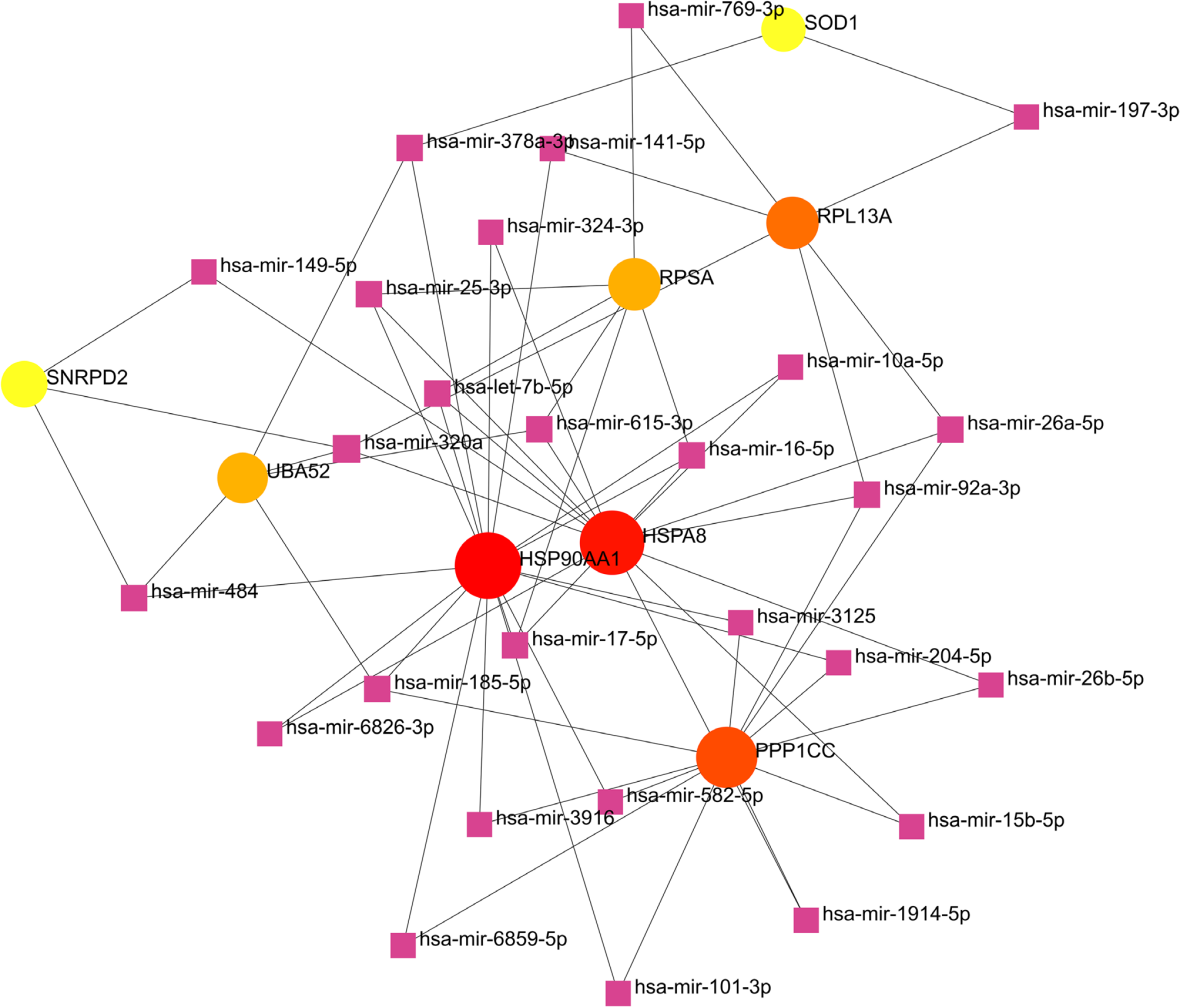
NetworkAnalyst generated an interconnected regulatory interaction network of miRNA-gene. The square nodes represent miRNAs, while the genes that interact with the miRNAs are depicted as circles. The size of the node area and the darkness of the color indicate their relative importance within the network

Transcription factors (TFs) are proteins capable of binding to gene-specific sequences [44], and miRNAs are a class of small non-coding RNA molecules [44]. Both can regulate gene expression. In our study, we separately analyzed interactions within the NetworkAnalyst platform (including ENCODE and MiRTarBase databases) to construct a TF-mRNA-miRNA interaction network. By analyzing the interaction networks of TF-mRNA-miRNA, we identified 46 key transcription factors that can regulate core genes. Among them, 13 TFs can regulate HSP90AA1, 12 TFs can regulate HSPA8, and 6 TFs can regulate SOD1. In the regulatory network, SREBF2, GATA2, and SRF are particularly important, as they can regulate both HSP90AA1 and HSPA8 (Fig. 7). Additionally, we obtained a total of 294 miRNAs that can regulate core genes. By applying a target connectivity criterion of ≥ 1 for miRNAs, we eventually identified the top 27 miRNAs. Among them, 17 miRNAs can regulate HSP90AA1, and 15 miRNAs can regulate HSPA8. Specifically, miR-320a can regulate HSPA8 and SNRPD2, miR-378a-3p can regulate HSP90AA1, and miR-26a-5p can simultaneously regulate HSPA8, PPP1CC, UBA52, and SOD1 (Fig. 8). The topological tables for the regulatory networks of TF-mRNA-miRNA are presented in Tables S7 and S8.

### Expression of core proteins and transcription factors in ovarian cancer

**Fig. 9:**
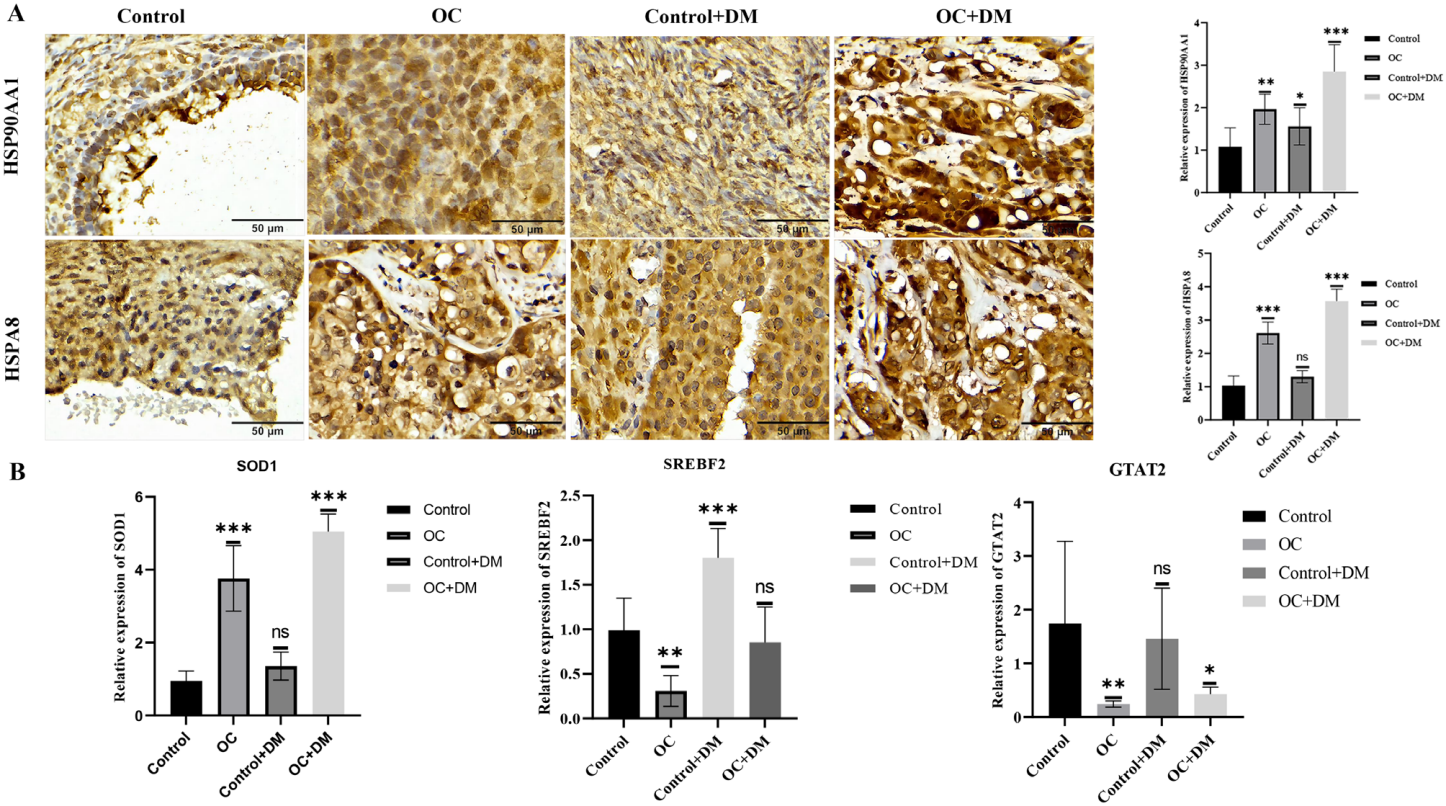
Expression of Core Proteins and Transcription Factors in Ovarian Tumors. **(A)** Immunohistochemical staining of HSP90AA1 and HSPA8 in age-matched high-grade serous ovarian tissues and non-malignant ovarian tumor tissues (*n* = 4). **(B)** Expression changes of core proteins HSP90AA1, HSPA8, and SOD1, as well as transcription factors SREBF2 and GTAT2 (*n* = 4). Control, Non-malignant ovarian tumor group; Control + DM, Non-malignant ovarian tumor group combined with DM; OC, High-grade serous ovarian cancer group; OC + DM, High-grade serous ovarian cancer group combined with DM. *p < 0.05, **p < 0.01, ***p < 0.001

We demonstrated differential expression of HSP90AA1 and HSPA8 in non-malignant ovarian tumor tissues, high-grade serous ovarian cancer samples, non-malignant ovarian samples concomitant with diabetes, and diabetes concomitant with high-grade serous ovarian cancer samples through Immunohistochemistry (IHC) and RT-qPCR. Additionally, we assessed the differential expression of the core protein SOD1 and the transcription factors SREBF2 and GTAT2 in these samples using RT-qPCR. The IHC and RT-qPCR results revealed that HSP90AA1 and HSPA8 were downregulated in non-malignant ovarian tumor tissues and significantly upregulated in high-grade serous ovarian cancer samples. Furthermore, there was no significant difference in non-malignant ovarian tumor tissues concomitant with diabetes, but a significant increase was observed in high-grade serous ovarian cancer samples (Fig. 9A, P < 0.001), consistent with our genetic analysis. Subsequently, we further evaluated the relationship between the expression of the core protein SOD1 and the transcription factors SREBF2 and GTAT2 in different samples. RT-qPCR results showed that SOD1 exhibited consistent expression with HSP90AA1 and HSPA8, while SREBF2 and GTAT2 were highly expressed in non-malignant ovarian tumor tissues and significantly decreased in high-grade serous ovarian cancer samples. Moreover, there was no significant difference in non-malignant ovarian tumor tissues concomitant with diabetes, but a significant decrease was observed in high-grade serous ovarian cancer samples (Fig. 9B, P < 0.001). In conclusion, these results suggest a significant increase in HSP90AA1 and HSPA8 concomitant with diabetes, and SREBF2 and GTAT2 may regulate the expression of HSP90AA1 and HSPA8.

## Discussion

The relationship between DM and OC is an area of research that has gained significant attention. Patients with diabetes often experience a chronic inflammatory state, which can lead to excessive secretion of inflammatory cytokines, growth factors, and interleukin-1 (IL-1) [45, 46]. Chronic inflammation may influence the growth, invasion, and metastasis of ovarian cancer cells by activating various inflammatory mediators and cytokines. Additionally, inflammation can impact ovarian cancer development through mechanisms such as modulation of the tumor microenvironment and immune escape [47, 48]. Hence, we explored potential interactions between monocytes in the OC microenvironment and PBMCs in individuals with DM from a unique perspective, aiming to identify common therapeutic targets.

In this study, we employed a network-based approach to investigate the gene expression profiles of two scRNA-seq datasets derived from OC and DM. Our analysis aimed to identify molecular targets that could serve as potential biomarkers for OC. Within the ovarian cancer scRNA-seq dataset, we identified eight major cell clusters. Moreover, analysis of the diabetes PBMC scRNA-seq dataset allowed us to identify three major cell clusters, with monocytes comprising a significant proportion of the cell clusters. Notably, our immune infiltration analysis of OC and DM RNA-seq data revealed substantial differences in monocyte infiltration between the two conditions. Monocytes are a central component to the innate immune response to pathogens [49]. There is literature indicating that in a mouse model with subcutaneous injection of human ovarian cancer cells, tumor volume significantly decreased in animals injected with interferon and monocytes at the early stage of tumor formation, and in some animals, tumors completely disappeared [50]. Stimulating the expression of CD44 in monocytes promotes apoptosis of ovarian cancer cells, favoring immune suppression [51]. Monocytes in the blood, besides their immune functions, also belong to a complex tissue control system (TCS). After simple and precise immunotherapy, stage IV ovarian cancer with liver metastasis completely regressed in the presence of diabetes [52]. Metformin downregulates the expression and ectonucleotidase activity of CD39 and CD73 on monocytes and various monocyte MDSC subpopulations in diabetic mice with OC, blocking the suppressive function of myeloid-derived suppressor cells (MDSC) and inducing apoptosis of ovarian cancer cells [53]. In non-obese diabetic-severe combined immunodeficiency (NOD-SCID) mice, monocytes modulated by bone marrow dendritic cells blocked with B7-H1 exhibited a more effective capacity to inhibit the growth of human ovarian cancer [54].

By identifying the overlapping differentially expressed genes in monocytes between DM and OC, we were able to determine 10 core genes with differential expression, indicative of their relevance to the pathogenesis of both conditions. These genes include HSP90AA1, HSPA8, SNRPD2, UBA52, SOD1, RPL13A, RPSA, ITGAM, PPP1CC, and PSMA5.Heat shock proteins (HSPs) play a protective role in shielding cells from oxidative stress, inflammation, and apoptosis [55]. Recent studies have indicated that activation of HSP90AA1 promotes tumor progression, invasion, and chemotherapy resistance [56]. HSP90AA1 acts as an extracellular secretory factor involved in inflammation, facilitating the malignant phenotype formation in tumor cells [57]. Inhibition of HSP90AA1 activity has been shown to reduce tumor necrosis factor (TNF) mRNA levels [58] and enhance glucose-stimulated insulin secretion and gene expression related to β-cell function [59]. Heat shock protein A8 (HSPA8) participates in cellular stress response, protein folding and assembly, protein transport, and protein degradation processes [60]. In the ovarian cancer tumor microenvironment, HSPA8 is implicated in modulating the immune response of monocytes/macrophages [61]. Furthermore, HSPA8 plays an important role in insulin secretion and insulin receptor signaling in DM [62]. Small nuclear ribonucleoprotein D2 (SNRPD2) consists of small nuclear ribonucleoproteins and small nuclear RNAs (snRNAs), playing a crucial role in the splicing process and gene expression regulation [63]. Aberrant expression or functional alterations of SNRPD2 in ovarian cancer may lead to splicing errors, affecting gene expression regulation in tumor cells [64]. UBA52 is a protein associated with ubiquitin, and its role has been reported in other pathologies. In traumatic brain injury, altered mRNA and protein levels of UBA52 have been observed [65]. Upregulated UBA52 has been found in the contexts of diabetic nephropathy and hepatoma cell apoptosis. Additionally, UBA52 deficiency in mice is associated with decreased protein synthesis, cell cycle arrest, and death during embryonic development [66]. Superoxide dismutase 1 (SOD1) regulates the levels of superoxide originating from the mitochondrial intermembrane space, cytosol, and peroxisome [67, 68]. Increased expression of SOD1 genes in animal models may decrease fasting blood glucose and hemoglobin A1c [69], and contribute to the survival of hypertrophied beta cells during chronic hyperglycemia. Conversely, genetic disruption of the SOD1 gene causes glucose intolerance and impairs beta cell function [70]. SOD1 is a critical determinant of platinum resistance in ovarian cancer and represents a target for overcoming this resistance [71].Small nucleolar RNAs (snoRNAs) are noncoding RNAs that form ribonucleoproteins involved in guiding covalent modifications of ribosomal and small nuclear RNAs in the nucleus. Loss of Rpl13a snoRNAs alters mitochondrial metabolism, reduces reactive oxygen species levels, increases glucose-stimulated insulin secretion from pancreatic islets, and enhances systemic glucose tolerance [72]. Previous studies have reported that under normoxic conditions, the most stably expressed genes in ovarian cancer cells are GAPDH/TBP, while under hypoxic conditions, the most stable candidate housekeeping genes are RPL13A/SDHA [73]. ITGAM encodes the α chain of integrin αMβ2 (CD11b). CD11b forms the leukocyte adhesion molecule β2 integrin with CD18, known as macrophage differentiation antigen-1 (Mac-1) [74]. The expression of Mac-1 and ICIAM-1 in the proliferative diabetic retina suggests the involvement of adhesion molecules in the pathogenesis of diabetic microvascular complications [75]. Furthermore, under pathological conditions, Mac-1 serves as a key adhesion molecule that facilitates cancer progression and mediates the adhesion of tumor cells to the endothelium of blood vessels [76]. The PPPC family has been shown to play essential roles in tumor cell proliferation [77], metastasis [78], and resistance to chemotherapy [79]. Additionally, GYS1 and PPP1CC have been reported to improve insulin resistance by regulating miR-140-5p in diabetes [80].Proteasome alpha subunits (PSMAs) have been implicated in the malignant progression of various human cancers [81]. In survival analyses, PSMA1-7 showed significant prognostic value in breast, lung, and gastric cancer. Furthermore, potential correlations between PSMAs and survival outcomes have been observed in ovarian cancer, colorectal cancer, and melanoma using Kaplan‒Meier Plotter [82, 83].

With each new scientific discovery, the structure of the GO resource continually evolves to incorporate biological knowledge of gene functions and to constantly improve its ability to reflect the latest state of biological understanding [84]. GO analysis was performed using the “clusterProfiler” package, and the analysis involved three ontological categories processes: biological process (molecular activity), cellular component (gene regulatory function), and molecular function (activity at the molecular level), utilizing the GO database as a source of information. Among the top GO terms in the biological process category were positive regulation of neutrophil degranulation and neutrophil activation involved in the immune response. Neutrophils serve as the first responders to inflammation and infection, while monocytes belong to the neutrophil species. It has been reported in the literature that treatment of orthotopic mouse ovarian cancer tumors with an anti-TGFBI antibody reduced peritoneal tumor size, increased tumor monocytes, and activated β3-expressing unconventional T cells [85]. Furthermore, alleviating the symptoms and complications associated with diabetes can be achieved by inhibiting inflammatory monocyte infiltration and altering macrophage characteristics [86].In the cellular component analysis, two major GO pathways were identified: secretory granule lumen and cytoplasmic vesicle lumen. These pathways play crucial roles in the development and progression of ovarian cancer and diabetes, including regulation of intracellular substance storage, transport, transfer, invasion, and formation of drug resistance [87]. In the context of diabetes, they are involved in processes related to insulin secretion, insulin resistance, intracellular substance transport and secretion associated with glucose metabolism and insulin secretion regulation [88].According to the molecular function analysis, the top GO terms were GDP-dissociation inhibitor activity and MHC class II protein complex binding. In ovarian cancer, MHC class II protein complex binding is associated with immune surveillance and immune evasion. Reduced expression or dysfunctional MHC class II protein complex binding prevents effective recognition and elimination of ovarian cancer cells by the immune system, promoting tumor development and metastasis [89]. In diabetic mice on a high-fat diet, the MHC II immune peptidome underwent quantitative and qualitative changes, highlighting the link between glycation reactions and alterations in MHC II antigen presentation, which may contribute to the development of type 2 diabetes complications [90]. KEGG pathway enrichment analysis of candidate target genes revealed their association with 211 signaling pathways, among which the interleukin-17 (IL-17) signaling pathway is a well-known cancer signaling pathway [91]. There are research reports suggesting that IL-17 may specifically modulate inflammatory monocytes during the later phases of the inflammatory response [92].

We have also identified associations between diseases based on TF-genes and miRNA-genes interactions. TFs are proteins that bind to specific gene sequences, known as promoters, and regulate gene transcription and expression [44]. Extensive research has revealed the regulatory roles of several TFs in the pathogenesis of OC and DM [93]. Notably, SREBF2, GATA2, PPARG, NFIC, ELK4, RELA, E2F1, and SRF have been implicated as TFs associated with various types of OC. For instance, the downregulation of sterol regulatory element binding protein 2 (SREBF2) inhibits the serine protease 8 (PRSS8)/sodium channel epithelial 1alpha subunit (SCNN1A) axis, leading to reduced cell proliferation, migration, and epithelial-mesenchymal transition in OC [94]. Moreover, the GATA2 gene has been identified as a prognostic factor in stromal-related studies of colon cancer [95], and it has also been implicated as a molecular signature in ovarian cancer through a network medicine perspective [96]. Regarding the visualization of gene-miRNA interactions, miR-320a, miR-378a-3p, miR-26a-5p, miR-92a-3p, and miR-484 have been associated with the pathogenesis of OC. For example, miR-320a promotes the proliferation and invasion of epithelial ovarian cancer cells by targeting RASSF8 [97]. Additionally, decreased expression of miR-378a-3p has been closely linked to an unfavorable prognosis in ovarian cancer patients, as it inhibits cell proliferation and promotes apoptosis [98]. These findings contribute to our understanding of the connection between DM and OC.

## Conclusions

This study unravels potential mechanisms and common therapeutic targets in monocytes between OC and DM, shedding light on the pathogenesis of both diseases. Through bioinformatics analysis, immune infiltration analysis, and survival analysis, it was confirmed that the identified key targets could be crucial treatment targets. These findings may provide a basis for clinical application of targeted treatments in patients with concurrent ovarian cancer and diabetes mellitus.

### Electronic supplementary material

Below is the link to the electronic supplementary material.

**Supplementary Material 1:** Differentially expressed marker genes identified across 29 clusters in the GSE184880 dataset

**Supplementary Material 2:** Five clusters were categorized as monocytes in the GSE184880 dataset

**Supplementary Material 3:** Differentially expressed marker genes identified in the GSE165816 dataset

**Supplementary Material 4:** Ten clusters were categorized as monocytes in the GSE184880 dataset

**Supplementary Material 5:** GO functional enrichment assessment was conducted on the PPI network regulating diabetes and ovarian cancer

**Supplementary Material 6:** The top 10 enriched KEGG pathways of the differentially expressed proteins

**Supplementary Material 7:** The topological tables of TF genes

**Supplementary Material 8:** The topological tables of miRNA genes

**Supplementary Material 9:** Supplementary legends

## Data Availability

The study utilized publicly available datasets for analysis. Specifically, the single-cell databases GSE184880 and GSE165816, as well as transcriptome data from GSE40595 and GSE29142, were sourced from the GEO platform (https://www.ncbi.nlm.nih.gov/geo/).

## Abbreviations

DM: Diabetes mellitus
OC: Ovarian cancer
PPI: Protein‒protein interaction
GO: Gene Ontology
KEGG: Kyoto Encyclopedia of Genes and Genomes
IHC: Immunohistochemistry
RT: qPCR-Real-time quantitative PCR
PBMCs: Peripheral blood mononuclear cells
IGF: 1-Insulin growth factor-1
scRNA: seq-Single-cell RNA sequencing
TME: Tumor microenvironment
GEO: Gene Expression Omnibus
NCBI: National Center for Biotechnology Information
DEGs: Differentially expressed genes
MF: Molecular function
CC: Cellular components
BP: Biological process
OS: Overall survival
DAB: 3,3’-diaminobenzidine
TFs: Transcription factors
IL: 1-Interleukin-1
TCS: Tissue control system
MDSC: Myeloid-derived suppressor cells
HSPs: Heat shock proteins
TNF: Tumor necrosis factor
HSPA8: Heat shock protein A8
SNRPD2: Small nuclear ribonucleoprotein D2
snRNAs: Small nuclear ribonucleoproteins and small nuclear RNAs
SOD1: Superoxide dismutase 1
snoRNAs: Small nucleolar RNAs
CD11b: αchain of integrin αMβ2
Mac: 1-Macrophage differentiation antigen-1
PSMAs: Proteasome alpha subunits
IL: 17-Interleukin-17
SREBF2: Sterol regulatory element binding protein 2
PRSS8: Serine protease 8
SCNN1A: Sodium channel epithelial 1alpha subunit
