# Supplementary material for: Single-cell and transcriptomic analyses reveal the influence of diabetes on ovarian cancer
Source: BMC Genomics. 2024 Jan 2;25:1. doi: 10.1186/s12864-023-09893-2 (PMC10759538; doi:10.1186/s12864-023-09893-2)
Supplement: Supplementary file 9 — Supplementary Material 9: Supplementary legends [file 12864_2023_9893_MOESM9_ESM.docx]

**Supplementary Legends**

Additional file 1: Table S1. Differentially expressed marker genes identified across 29 clusters in the GSE184880 dataset. Table S2. Five clusters were categorized as monocytes in the GSE184880 dataset. Table S3. Differentially expressed marker genes identified in the GSE165816 dataset. Table S4. Ten clusters were categorized as monocytes in the GSE184880 dataset. Table S5. GO functional enrichment assessment was conducted on the PPI network regulating diabetes and ovarian cancer. Table S6. The top 10 enriched KEGG pathways of the differentially expressed proteins. Table S7. The topological tables of TF genes. Table S8. The topological tables of miRNA genes.
